# Correction: Structure-based discovery of fiber-binding compounds that reduce the cytotoxicity of amyloid beta

**DOI:** 10.7554/eLife.01252

**Published:** 2013-07-23

**Authors:** Lin Jiang, Cong Liu, David Leibly, Meytal Landau, Minglei Zhao, Michael P Hughes, David S Eisenberg

Jiang L, Liu C, Leibly D, Landau M, Zhao M, Hughes MP, Eisenberg DS. 2013. Structure-based discovery of fiber-binding compounds that reduce the cytotoxicity of amyloid beta. *eLife* **2**:e00857. doi: http://dx.doi.org/10.7554/eLife.00857. Published 16 July 2013

The affiliation contained spelling errors. It should read:

Departments of Chemistry and Biochemistry and Biological Chemistry, Howard Hughes Medical Institute, UCLA–DOE Institute for Genomics and Proteomics, University of California, Los Angeles, Los Angeles, United States

In Table 3, the contents of rows 7 and 8 of the ‘Description’ column were reversed.

The article has been corrected accordingly.

